# The draft genome of the Temminck’s tragopan (*Tragopan temminckii*) with evolutionary implications

**DOI:** 10.1186/s12864-023-09857-6

**Published:** 2023-12-07

**Authors:** Xuejuan Li, Xiaoyang Wang, Xiaoping Yu, Chao Yang, Liliang Lin, Yuan Huang

**Affiliations:** 1 College of Life Sciences, Shaanxi Normal University, Xi’an, China; 2 School of Biological and Environmental Engineering, Xi’an University, Xi’an, China; 3 Shaanxi Institute of Zoology, Xi’an, China

**Keywords:** Temminck’s tragopan, Genome feature, Comparative genomics, Positive selection

## Abstract

**Background:**

High-quality genome data of birds play a significant role in the systematic study of their origin and adaptive evolution. The Temminck’s tragopan (*Tragopan temminckii*) (Galliformes, Phasianidae), a larger pheasant, is one of the most abundant and widely distributed species of the genus *Tragopan,* and was defined as class II of the list of national key protected wild animals in China. The absence of a sequenced genome has restricted previous evolutionary trait studies of this taxa.

**Results:**

The whole genome of the Temminck’s tragopan was sequenced using Illumina and PacBio platform, and then de novo assembled and annotated. The genome size was 1.06 Gb, with a contig N50 of 4.17 Mb. A total of 117.22 Mb (11.00%) repeat sequences were identified. 16,414 genes were predicted using three methods, with 16,099 (98.08%) annotated as functional genes based on five databases. In addition, comparative genome analyses were conducted across 12 Galliformes species. The results indicated that *T. temminckii* was the first species to branch off from the clade containing *Lophura nycthemera*, *Phasianus colchicus*, *Chrysolophus pictus*, *Syrmaticus mikado*, *Perdix hodgsoniae*, and *Meleagris gallopavo*, with a corresponding divergence time of 31.43 million years ago (MYA). Expanded gene families associated with immune response and energy metabolism were identified. Genes and pathways associated with plumage color and feather development, immune response, and energy metabolism were found in the list of positively selected genes (PSGs).

**Conclusions:**

A genome draft of the Temminck’s tragopan was reported, genome feature and comparative genome analysis were described, and genes and pathways related to plumage color and feather development, immune response, and energy metabolism were identified. The genomic data of the Temminck’s tragopan considerably contribute to the genome evolution and phylogeny of the genus *Tragopan* and the whole Galliformes species underlying ecological adaptation strategies.

**Supplementary Information:**

The online version contains supplementary material available at 10.1186/s12864-023-09857-6.

## Background

Birds are the one of the most species-rich monophyletic group of land vertebrates [1]. They are characterized by generally having smaller genome sizes ranging from 1 Gb to 2.1 Gb [2]. Compared with other vertebrates, avian genomes are relatively small with conserved synteny [3, 4] and few repetitive elements, which made them suitable species for use in evolutionary research [5]. With the advent of third-generation sequencing, it is now possible to provide reliable genome assembly, and currently, with PacBio and Oxford Nanopore being the most popular technologies to generate long reads for genome assembly [6]. The advancement in sequencing technologies have facilitated easier generation of genome data of avian species. The genome sequences of birds, together with the collection of their morphological, physiological, ecological, and behavioral traits, could provide information on studies of evolution, ecology, population genetics, neurobiology, development, and conservation [7]. For example, avian genomic data were significant for understanding the origin and evolution of traits, and increased availability of genome sequences have facilitated studies on the evolution of powered flight, body size variation, beak morphology, plumage coloration, high-elevation colonization, migration, and vocalization [5], as well as aspects of phylogenetic evolution and adaption [3, 8], transposable elements (TEs) [1], vocal learning [9], and genome size evolutionary dynamics [2].

The genus *Tragopan* belongs to the family Phasianidae (Galliformes), and consists of five species—*T. melanocephalu*s, *T. satyra*, *T. blythii*, *T. temminckii*, and *T. caboti* [10]. The Temminck’s tragopan (*T. temminckii*), a larger pheasant, is one of the most abundant and widely distributed species of the genus *Tragopan* [11], and was defined as class II of the list of national key protected wild animals in China [12]. It is mainly distributed in southwest areas of China [13], including provinces of Sichuan, Yunnan, Xizang and Chongqing, along with adjacent regions of India, Myanmar, and Vietnam [14, 15]. This species occurs at an elevation of 1000–3500 m [13]. The Temminck’s tragopan is an omnivorous bird, feeding primarily on plant matter [16]. Previous studies found that this species feeds chiefly on herbs and ferns in spring and winter, and mature fruits in summer and autumn [17].

Previous studies related to the Temminck’s tragopan have been mainly concentrated on behavioral and ecological investigations, such as the activity rhythm [18, 19], niche [20], diet [11, 17], flocking behavior [21], habitat [22, 23], and breed [24]. In addition, previous research has shown that the karyotype of *T. temminckii* consisted of a diploid number of chromosomes of 80, with seven pairs of macrochromosomes and 33 pairs of microchromosomes [25]. Phylogenetically, *Tragopan* is a part of the clade containing other large pheasants, *Lophophorus* and *Tetraophasis*, based on analysis of many different data types, such as mitochondrial genomes [26–28], mitochondrial and nuclear DNA sequence data [29], and ultraconserved elements (UCEs) [30]. This clade of *Tragopan*/*Lophophorus*/*Tetraophasis* is sister to the larger clade containing *Chrysolophus*, *Phasianus*, *Lophura*, *Crossoptilon*, *Syrmaticus, Perdix*, *Pucrasia*, *Bonasa* and *Meleagris* [26–28]. In terms of adaptation and evolutionary history, to date, only a limited number of studies have been performed, and consequently, comprehensive genetic analyses based on large-scale genomic data are necessary to understand their ecological and evolutionary adaptations.

Sexually dimorphic plumage coloration is common in birds, with plumage in males brighter than females, a phenomenon that is associated with environmental constraints, sexual selection or intraspecific competition between males and females [31]. The male Temminck’s tragopan is a brilliantly colored bird, however, the genetic basis of plumage color that lead to this gorgeous coloration is absent. Plumage color, a highly polygenic trait, can be affected by multiple coding genes, regulatory genes, and gene–gene epistasis interactions [32]. To date, several genes associated with the plumage color in birds have been reported, such as *MC1R*, *MITF*, *ASIP*, *TYRP1*, and *BCO2* [31, 33, 34]. Some candidate genes, such as *MITF*, *EDNRB2*, *TBC1D22A*, *EDA*, *SLC45A2*, and *GOLGB1*, have been identified to be related to avian plumage color based on genome-wide analyses [35, 36]. The genome of the Temminck’s tragopan could help uncover whether any of these genes are essential for the development of coloration in this species.

In this study, a de novo assembled genome of the Temminck’s tragopan was obtained from a combination of PacBio long reads and Illumina short reads. Comparative genomic analyses of the Temminck’s tragopan were carried out with the other 11 other available Galliformes species. With genome-scale insights, the genomic characteristic, phylogeny, and evolution of the Temminck’s tragopan were investigated, and candidate genes and signaling pathways associated with plumage color and feather development, immune response, and energy metabolism were explored. The assembled genomic resource of the Temminck’s tragopan will benefit researchers in future studies on their genetics and ecological evolution.

## Results

### Genome assembly and characteristic

In total, 93.71 Gb raw data of Illumina platform were generated, with a depth of 88.41 × (Table S1), while 3,271,105 reads of PacBio platform were obtained, totaling 29.73 Gb. The genome size of the Temminck’s tragopan was estimated at 1.06 Gb, with a GC content of 42.09%. The contig N50 was 4.17 Mb, with the largest contig of 16.6 Mb. In total, the gene annotation predicted 16,414 genes across the genome. The average length of genes was 19,220.68 bp, with an average length of 161.49 bp in exons and 1,716.97 bp in introns, respectively. A total of 11,494 genes were supported by three predicting methods (Fig. S1a). In addition, non-coding RNAs predicted results identified 200 miRNAs belonging to 95 families, 129 rRNAs of four families, and 289 tRNAs of 23 families, respectively. A total of 283 pseudogenes were also found. The contamination assessment result of assembly genome of the Temminck’s tragopan showed that 90.90% terms were corresponding to Chordata, which represented almost no contamination (Fig. S1b).

**Fig. 1:**
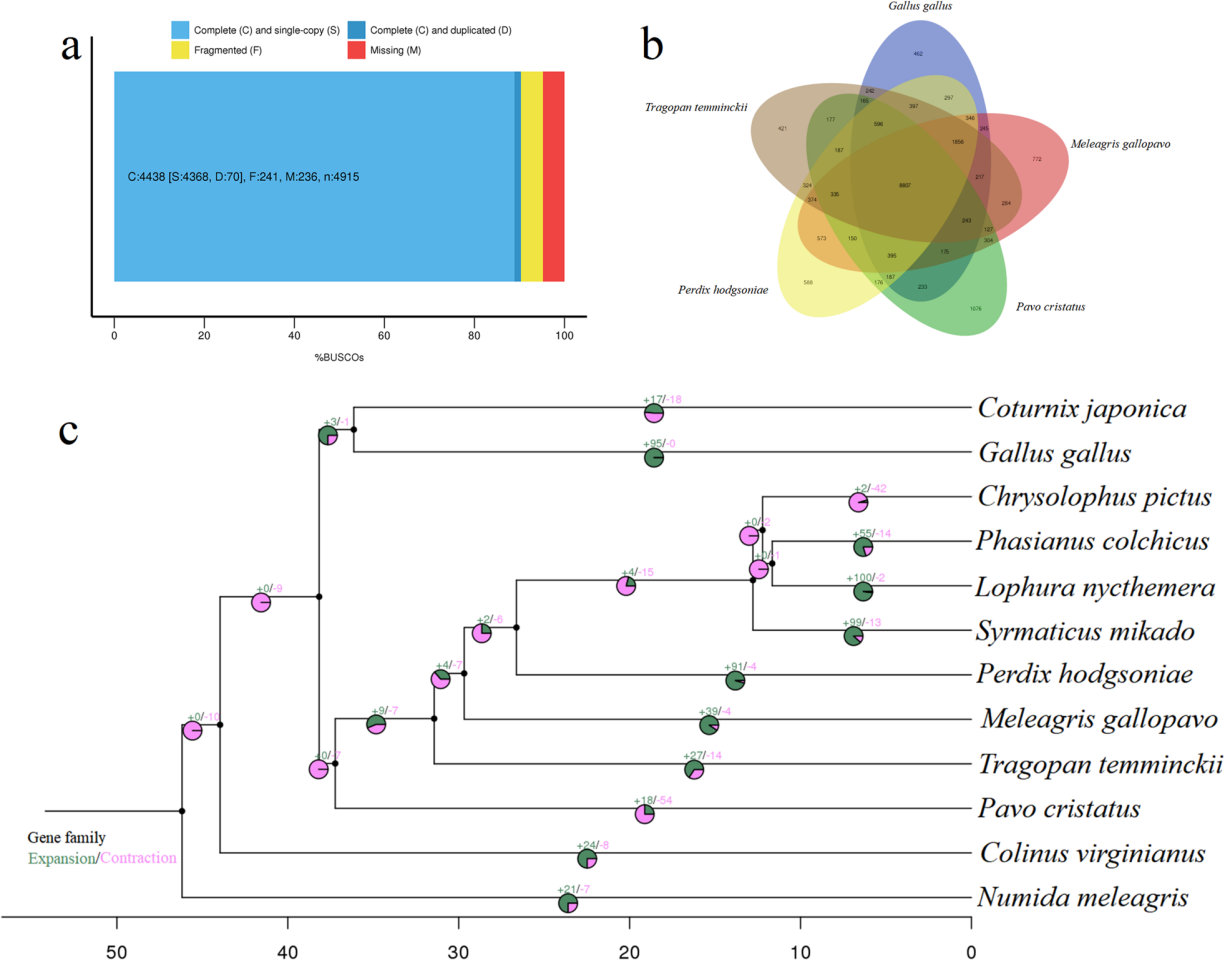
The assembly assessment in the Temminck’s tragopan, and comparative genomic analysis among Galliformes species. **a** BUSCO assessment result. **b** Venn diagram of gene families among five closely related species (*Perdix hodgsoniae*, *M. gallopavo*, *T. temminckii*, *Pavo cristatus*, and *G. gallus*). **c** the expanded and contracted gene families of 12 sampled Galliformes species, with green represented gene families that experienced expansion events, and pink represented gene families under contraction

BUSCO analysis evaluated the genome assembly completeness and showed that 4,438 complete BUSCOs (90.30%) were identified, including 4,368 single-copy (88.87%), and 70 duplicated BUSCOs (1.42%) (Fig. 1a). 241 BUSCOs (4.90%) were fragmented, and 236 BUSCOs (4.80%) were missing (Fig. 1a). Besides, 427 CEGs (93.23%) were identified in the CEGMA database, and 224 out of 248 highly conserved CEGs were found accounting for 90.32%. Furthermore, 99.01–99.89% of Illumina clean reads from five libraries were mapped to the assembly, with 91.20–96.09% properly mapped. These results suggested that assembled genome sequences of the Temminck’s tragopan were relatively complete and high-quality genome data.

Repeats percentage was 11.00% with a total length of 117.22 Mb in the Temminck’s tragopan. Long interspersed nuclear elements (LINEs) accounted for most repeats, occupying about 8.11%, while the proportion of short interspersed nuclear elements (SINEs) was 0.02% (Table S2). In addition, the total length of 1,685,298 bp was classified as simple sequence repeats (SSRs), with a proportion of 0.16% (Table S2).

### Gene function

**Fig. 2:**
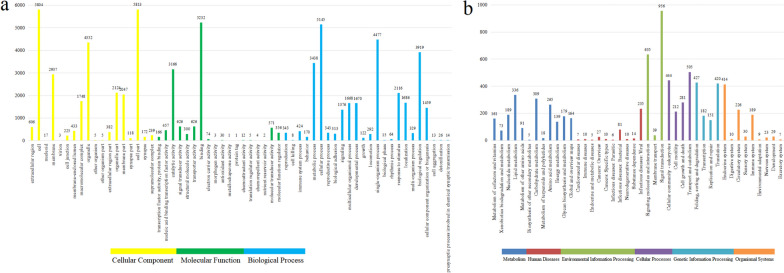
Functional annotation result of the assembly genome in the Temminck’s tragopan. **a** the GO database. **b** the KEGG database

Overall, a total of 16,099 (98.08%) genes were functionally annotated based on five databases. Collectively, 9,260 (56.42%) out of 16,414 total gene sequences were functionally annotated using the GO database, as well as 9,963 genes (60.70%) from the KEGG, 11,642 genes (70.93%) from the KOG, 16,040 genes (97.72%) from the TrEMBL, and 16,074 genes (97.93%) from the NR database, respectively. The distributions of GO terms within three main GO domains including the cellular component (CC), molecular function (MF), and biological process (BP) are presented in Fig. 2a, with details of mainly GO terms showed in Table S3. GO term numbers in the Temminck’s tragopan were similar to other birds [37].

KEGG functional annotation results suggested that these processes were found in six sections, including metabolism (1,434 genes), human diseases (323 genes), environmental information processing (1,448 genes), cellular processes (1,189 genes), genetic information processing (1,132 genes), and organismal systems (757 genes), in which corresponding lipid metabolism (336 genes), infectious diseases: viral (235 genes), signal transduction (956 genes), transport and catabolism (505 genes), folding, sorting and degradation (427 genes), and endocrine system (414 genes) the highest term, respectively (Fig. 2b).

Family classification results showed that a total of 21,803 gene families were identified, with 5,846 gene families commonly shared among 12 sampled Galliformes species (Fig. S2a). The number of species-specific gene families ranged from one in *Chrysolophus pictus* to 302 in *Pavo cristatus*, with eight in *T. temminckii* (Fig. S2a)*.* Further analyses showed that 8,807 gene families were commonly shared in closely related five species, including *Perdix hodgsoniae*, *M. gallopavo*, *T. temminckii*, *Pavo cristatus*, and *G. gallus* (Fig. 1b)*.*

### Phylogeny and divergence time

The resulting phylogenetic tree was supported by relatively higher bootstraps, with values of 100 in all nodes except for 99 of the node formed by *Lophura nycthemera* and *Phasianus colchicus* (Fig. S2b). The reconstructing result recovered the monophyly of Phasianidae, and phylogenetic relationships of ((((((((((*L. nycthemera* + *Phasianus colchicus*) + *Chrysolophus pictus*) + *Syrmaticus mikado*) + *Perdix hodgsoniae*) + *M. gallopavo*) + *T. temminckii*) + *Pavo cristatus*) + (*Coturnix japonica* + *G. gallus*)) + *Colinus virginianus*) + *Numida meleagris*) was supported (Fig. S2b). The phylogenetic analysis supported that the sampled Phasianidae taxa were divided into two major groups, with group I consisting of two species including one partridge (*Coturnix japonica*) and one pheasant (*G. gallus*), group II including two partridges (*Perdix hodgsoniae* and *M. gallopavo*) and six pheasants, respectively (Fig. S2b). *T. temminckii* was the first species to branch off from the clade containing *L. nycthemera*, *Phasianus colchicus*, *Chrysolophus pictus*, *S. mikado*, *Perdix hodgsoniae*, and *M. gallopavo*.

The divergence time between Phasianidae and Odontophoridae was 43.96 million years ago (MYA) (95% highest posterior density (HPD) = 38.43–50.19 MYA) (Fig. S2c). The divergence times of genera within Phasianidae ranged from 11.65 MYA between *L. nycthemera* and *Phasianus colchicus* to 38.16 MYA between the clade of *Coturnix japonica*/*G. gallus* and other sampled Phasianidae species (Fig. S2c), corresponding to Miocene and Eocene. *T. temminckii* diverged from the recent common ancestor approximately 31.43 MYA with 95% HPD = 28.17–36.46 MYA (Fig. S2c), corresponding to Oligocene.

The Temminck’s tragopan genome showed high synteny with the chicken genome, with contig00419 and contig00124 in the Temminck’s tragopan mapped to chromosome 4 in chicken, and contig00295 and contig02393 mapped to chromosome 5, respectively (Fig. S3).

### Expansion and contraction

Comparative genomic investigation among 12 sampled Galliformes indicated that 27 expanded gene families and 14 contracted gene families were identified in the Temminck’s tragopan (Fig. 1c). The functional identities presented in GO terms are shown in Fig. S4a, with the main GO terms exhibited in Table S3. Among GO functional enrichment results, several significantly expanded gene families were involved in the immune response including defense response to virus (GO:0051607) and immune response (GO:0006955) in the BP section, and energy metabolism containing fatty acid biosynthetic process (GO:0006633) in the BP section, and oxidoreductase activity (GO:0016491), GTPase activity (GO:0003924) and GTP binding (GO:0005525) in the MF section.

In addition, in the functional KEGG enrichment, the expanded gene families were identified with the immune response involving drug metabolism—other enzymes (ko00983), regulation of autophagy (ko04140), RIG-I-like receptor signaling pathway (ko04622), MAPK signaling pathway (ko04010), Toll-like receptor signaling pathway (ko04620), Jak-STAT signaling pathway (ko04630), and cytokine-cytokine receptor interaction (ko04060). These immune-related pathways have been also found in previous studies based on avian genomes, such as the MAPK signaling pathway [37], which may be related to response to the environment, and could enhance their adaptations. These unique expansions of GO and KEGG enrichment in the Temminck’s tragopan might be important for the ability of immune response and energy metabolism for their ecological environmental adaptation*.*

### PSG analysis

The results indicated that a total of 2,022 genes were found under positive selection in the Temminck’s tragopan. The functional enrichment analysis presented in the GO section showed in Fig. S4b, with main GO terms showed in Table S3. The top 10 significant GO terms were shown in Fig. S5, which revealed that the integral component of Golgi membrane (GO:0030173), ATP-dependent helicase activity (GO:0008026), and binding of sperm to zona pellucida (GO:0007339) were the most significant GO terms in a corresponding CC, MF, and BP section, respectively (Fig. S5a-c). While the phosphatidylinositol signaling system (ko04070) was the most significant KEGG term (Fig. S5d).

Functional annotation results of PSGs in the Temminck’s tragopan identified pathways and genes related to plumage color and feather development, immune response, and energy metabolism. The contents related to plumage color and feather development were identified, including melanocyte differentiation (GO:0030318), hair follicle morphogenesis (GO:0031069), hair follicle development (GO:0001942), keratinocyte differentiation (GO:0030216) in the BP section in GO terms, and MAPK signaling pathway, Notch signaling pathway and melanogenesis pathway in the KEGG (Table S4). Nine PSGs including *WNT10A*, *DVL1*, *ADCY5*, *MAPK1*, *CBP*, *tcf7l1*, *GNAO1*, *CAMK2G* and *EP300* were functional candidate genes involved in the melanogenesis pathway (ko04916), while 39 PSGs involved in the MAPK signaling pathway (ko04010) were identified (Table S5). Twelve candidate genes associated with plumage coloration were identified, such as *SLC45A2* (Table 1). The 362 position of amino acid Q in the *SLC45A2* gene was under significant positive selection effects (prob = 0.958).

**Table 1:** Candidate genes associated with plumage coloration in PSGs in the Temminck’s tragopan

Gene ID	Gene
EVMG011321.1	*PDGFRA*
EVMG001445.1	*SLC6A6*
EVMG001495.1	*CHCHD6*
EVMG001503.1	*RPN1*
EVMG005723.1	*PSMD6*
EVMG004808.1	*BTK*
EVMG013095.1	*Cnot6l*
EVMG011466.1	*SMAD6*
EVMG000561.1	*MAPKAPK2*
EVMG007178.1	*PBRM1*
EVMG008392.1	*TTBK2*
EVMG011707.1	*SLC45A2*

As functional identities presented, several immune-related contents were found in the Temminck’s tragopan, such as fibroblast growth factor binding (GO:0017134) in the MF section, and defense response to virus (GO:0051607) in the BP section of the GO result, and mTOR signaling pathway (ko04150) in the KEGG (Table S4). Thirty immune-related KEGG pathways was found, such as MAPK, Jak-STAT, and Toll-like receptor signaling pathway (Table S4). Among them, 17 PSGs (e.g., *MTOR*, *EGFR*, *PIK3R2*, *MAPK1*, and *CAMK2G*) were identified as involved in the ErbB signaling pathway.

Genes related to energy metabolism were also identified in the Temminck’s tragopan, such as mitochondrion (GO:0005739) in the CC section, ATPase activity (GO:0016887) in the MF section, and metabolic process (GO:0008152) in the BP section of GO terms, respectively, as well as oxidative phosphorylation (ko00190) in the KEGG (Table S4).

## Discussion

### Genome feature and evolution

The genome size of the Temminck’s tragopan (1.06 Gb) was similar to other Galliformes species reflected by the previous studies, ranging between 0.93 Gb of *Coturnix japonica* to 1.25 Gb of *Colinus virginianus* [8]. The GC content of the Temminck’s tragopan (42.09%) was within the ranges of other Galliformes species from 40.95% of *Odontophorus gujanensis* to 42.66% of *Colinus virginianus* [8]. In total, the predicted gene number of the Temminck’s tragopan (16,414) was within ranges of total gene numbers of other Galliformes species, from 15,429 of *Alectura lathami* to 17,883 of *Gallus gallus* reported for the previous genome assembly [8]. The proportion of complete BUSCOs of the Temminck’s tragopan (90.30%) was similar to that of other Galliformes species ranging from 89.6% of *A. lathami* to 94.9% of *G. gallus* [8].

The divergence time between Phasianidae and Odontophoridae (43.96 MYA) was similar to that of previous studies based on a combination of mitochondrial and nuclear data, such as 42.8 MYA [38] and 53.6 MYA [29], UCEs (39.9 MYA) [30], and genome data (46.46 MYA) [37]. Divergence times of genera within Phasianidae (11.65 MYA-38.16 MYA) were consistent with the previous study [39]. The divergence time of *Tragopan* divided with other closely related Phasianidae species (31.43 MYA) was similar to previous studies [28, 29], however, was earlier than that of the results of a combination of mitochondrial and nuclear data, and UCEs [30, 38].

### Plumage color and feather development

Traits, such as plumage coloration, usually have lineage specificity, are commonly related to selection pressure, and reflect the adaptive characteristics of species [5]. Major changes in key genes or at the genomic level may drive plumage color differentiation on a larger evolutionary scale [5]. The plumage color of chicken is a complex trait controlled by several genes [40]. Research related to plumage color has been widely studied in birds based on different data types, such as transcriptomes and genomes, and identified several significant signaling pathways and genes [5, 36, 41–43].

For related signaling pathways on plumage coloration, several confirmed pathways involved in pigmentation, such as the BMP signaling pathway, and pathways of cAMP, SCF-KIT, Notch, ERK, CREB/MITF/tyrosinase, Wnt/β-catenin and MAPK, and genes, such as *MC1R*, *TYR*, *SLC24A5*, and *DCT*, played a role in melanin proportion synthesis [41]. Some signaling pathways and genes were commonly found in previous investigations of birds, such as the *TYR* gene in chicken [44], the crested ibis [45], and ducks [46]. The melanogenesis pathway found in PSGs of the Temminck’s tragopan have also been identified in other birds [32, 41, 47–50], such as the chicken based on transcriptome [50] and genomic SNP data [32]. For genes enriched in melanogenesis pathway in PSGs of the Temminck’s tragopan, a previous study has been showed that miR-193b might participate in the adjustment of coat color in the skin tissue of Cashmere goats by targeting *WNT10A* and *GNAI2* [51]. Furthermore, some other genes were also found enriched in the melanogenesis pathway in previous studies, such as *TYRP1* [48] and *ASIP* [41]. In addition, for genes enriched in the MAPK signaling pathway in PSGs of the Temminck’s tragopan, *MAPK1* is the main member of MAPKs. By comparing transcription and protein levels between the red and black skin of *Plectropomus leopardus*, *ERK1/2* (corresponding to the *MAPK1* gene) was interfered with after RNAi, and the local skin of the tail would turn black [52].

For related genes on plumage coloration, more than 200 genes associated with pigmentation have been found in mammals, and genes associated with plumage color mutations were increasingly identified in birds [43]. Candidate genes related to plumage coloration found in PSGs of the Temminck’s tragopan have been also found in the other birds, such as *MAPKAPK2* [53], *SLC6A6* and *SMAD6* [49]. The *PDGFRA* gene found in PSGs of the Temminck’s tragopan was identified as associated with the coat color of mammals, such as cattle and goats. For example, genes (*e.g., PDGFRA* and *MITF*) were promising candidates for black and teat color in Holstein cattle [54]. According to previous studies, the *PDGFRA* gene was related to a proportion of black [54], *SLC6A6*, *CHCHD6*, *RPN1*, and *PSMD6* genes were associated with white plumage, *BTK*, *Cnot6l*, and *SMAD6* genes were correlated with grey plumage [49], *MAPKAPK2*, *PBRM1*, and *TTBK2* genes were associated with melanogenesis [53], and the *SLC45A2* gene played an important role in vesicle sorting in the melanocytes [42], respectively. Among them, the *SLC45A2* gene found in PSGs of the Temminck’s tragopan encoded a transporter protein, which mediated melanin synthesis, and has been also reported as related to plumage color in birds [36, 42, 55]. Besides, the *SLC45A2* gene played a significant role in vesicle sorting in the melanocytes, and two independent missense mutations (Tyr277Cys and Leu347Met) of this gene were associated with the silver plumage color in chicken [42]. Furthermore, the *SLC45A2* gene was also found to be positively selected in *Machaeropterus deliciosus*, and may explain its unique reddish-brown body plumage among other studied manakins [36]. The identified protein-coding and cis-regulatory mutations in *TYRP1*, *SOX10*, and *SLC45A2* underlay classical color phenotypes of pigeons [56].

### Immune response

The immune system was an interactive network of lymphoid organs, cells, humoral factors, and cytokines [57]. Multiple immune-related pathways have been also found in previous studies based on avian genomes, such as toll-like receptors (TLRs) [58, 59] and the MAPK signaling pathway [37], which play a significant role in their immune responses against infections. TLRs and Toll-like receptor signaling pathway were found in PSGs in the Temminck’s tragopan. TLRs, one family of functional genes, are present in nearly all multicellular organisms [60], and play a crucial role in the recognition of pathogens and activation of the immune system [59]. TLRs can be a possible thread linking hypoxia and venous thromboembolism (VTE) by recognition of damage-associated molecular patterns (DAMPs) generated by hypoxia [61]. Based on the reference genome and whole-genome resequencing population data, the results showed that the *TLR* gene diversity was low in *Thinornis novaeseelandiae*, and formed two distinct captive and wild genetic clusters [59]. The MAPK signaling pathway identified in PSGs in the Temminck’s tragopan was similar to previous genome data also including several related genes under positively selected in this pathway [37, 62, 63]. In addition, ErbB signaling pathway has been found significantly enriched in ducks based on genome-wide analyses, in which the candidate gene (*ABL1*) was enriched [64]. The ErbB and Wnt signaling pathway were also identified in genome data in the Chinese monal [63]. Consequently, the positive signals of genes and pathways identified in the Temminck’s tragopan may indicate adaptation to the ecological environment.

## Conclusions

In this study, the assembled and annotated genome of the Temminck’s tragopan (*T. temminckii*) was obtained by utilizing long-read PacBio and short-read Illumina sequencing. The genome characteristics, such as the genome size and repeat sequence, were reported, and comparative genomic studies were analyzed among sampled 12 Galliformes species. The results indicated that the genome size of the Temminck’s tragopan was 1.06 Gb, with 11.00% repeat sequences. *T. temminckii* was the first species to branch off from the clade containing *L. nycthemera*, *Phasianus colchicus*, *Chrysolophus pictus*, *S. mikado*, *Perdix hodgsoniae*, and *M. gallopavo*, with a corresponding divergence time of 31.43 MYA. Multiple genes and pathways related to plumage color and feather development, immune response, and energy metabolism were identified in the Temminck’s tragopan, such as the *SLC45A2* gene, melanocyte differentiation, MAPK signaling pathway, and lipid metabolic process, and which may be significant with their ecological adaptation. This genome will serve as an important resource to increase our knowledge of genome data in Galliformes birds, and will provide a deeper insight into their genomic characteristics and significance for avian evolution.

## Materials and methods

### Sample collection and sequencing

The muscle sample of male *Tragopan temminckii* was collected from a captive breeder of Lantian, Xi’an, Shaanxi Province, China in 2017, and preserved in the College of Life Sciences of Shaanxi Normal University with the voucher of LGJZ01 for muscle tissues. The genomic DNA was extracted using the CTAB method [65], while total RNA was extracted with TRIzol reagent following the recommended protocol provided by the manufacturer. DNA and RNA quality was checked using a combination of a NanoDrop 2000, Qubit 2.0, and Agilent 2100. DNA qualified with a DNA integrity number (DIN) and RNA with RNA integrity number (RIN) scores larger than 8.0, and OD260/280 ranged between 1.8 and 2.2 were used for the library preparation and construction. Short reads were sequenced on the Illumina NovaSeq 6000 platform, employing five small libraries (three of 270 bp and two of 350 bp), while long reads were obtained on the PacBio Sequel platform. The standard protocol of library preparation and sequencing method were used, consistent with previous studies [37, 66]. For RNA sequencing, data were obtained from the Illumina pipeline, and rRNA was isolated from total RNAs to contribute RNA fragment libraries, then fragmented randomly. The first-strand cDNA was synthesized using random hexamer primers, and the second-strand cDNA was synthesized using DNA polymerase I and RNase H. After the processing of end-repair, A-tail, adaptor ligation, and purification, PCR amplification was carried out.

### De novo genome assembly and assessment

The short- and long-reads were used to assemble the genome. Firstly, low-quality sequences and short-length reads of the PacBio raw data were filtered out, with quality value larger than 0.75 and length larger than 100 bp. LoRDEC v. 0.7 [67] was employed to correct long-read PacBio sequencing data using Illumina data. Subsequently, the short-read Illumina sequencing data were assembled preliminarily with platanus v. 1.2.4 [68] using default parameters. Finally, the dbg2olc [69] was run for mixed assemblies with corrected PacBio data and Illumina assembled results, using default parameters.

The contamination of the contig-level genome was assessed using the blobtools pipeline v. 0.9.19 [70, 71], by generating taxon annotated GC content coverage plots. Each contig was annotated based on blastn v. 2.2.31 [72], searching against the NCBI nucleotide database (nt, downloaded October 13, 2017). Besides, bwa-mem v. 0.7.7 [73] was employed for the whole Illumina raw reads mapped to the contig-level genome to calculate average coverage for per contig, with default parameters. The BAM results were sorted using samtools v. 1.3 [74], and passed to blobtools along with the blastn results.

Three methods were used to assess the quality of the genome assembly, including Benchmarking Universal Single-Copy Orthologs (BUSCO) evaluation, Core Eukaryotic Genes Mapping Approach (CEGMA) analysis, and reads remapping. Among them, BUSCO v. 2.0 [75] was employed to estimate the completeness of the de novo assembled genome draft by searching for 4,915 universal avian single-copy orthologs (aves_odb09), running under the genome mode to compute the proportion of complete, fragmented, and missing genes across the dataset. CEGMA v. 2.5 [76] was also used, with 458 CEGs. To evaluate the mapping rate of reads, the Illumina short reads were realigned to genome assembly.

### Genome annotation

To identify repeat regions of the assembled genome, the software LTR-FINDER v. 1.05 [77], MITE-Hunter [78], RepeatScout v. 1.05 [79], and PILER-DF v. 2.4 [80] were used to construct a repeat library based on the structure-based and de novo predictions, using default parameters. PASTEClassifier [81] was employed to classify this repeat library with default parameters. Then the repeat library was combined with the Repbase [82] database to build a final library. Finally, RepeatMasker v. 4.0.6 [83] was employed to annotate the repeat sequences based on the constructing repeat library, with parameters of ‘-nolow -no_is -norna -engine wublast -qq -frag 20,000’.

The assembled genome was annotated through three methods, including de novo, homologous, and RNA-seq gene annotation. For de novo gene prediction, Augustus v. 2.4 [84], Genscan [85], GlimmerHMM v. 3.0.4 [86], GeneID v. 1.4 [87], and SNAP v. 2006-07-28 [88] were used with default parameters. For homologous gene annotation, GeMoMa v. 1.3.1 [89] was employed using parameters of ‘-percent 0.95 -maxintron 20,000 -eachtranscript 10 -e 0.00001’, with referring protein sequences of the following five species: chicken, wild turkey, zebra finch, collared flycatcher, and great tit. For the RNA-seq method, Hisat v. 2.0.4 [90] and StringTie v. 1.2.3 [91] were used for assembly based on transcripts, using default parameters. TransDecoder v. 2.0 (available online: https://transdecoder.github.io/) and GeneMarkS-T v. 5.1 [92] were employed for gene prediction, employing default parameters. PASA v. 2.0.2 [93] was used to predict unigene sequences with non-reference assembly based on transcriptome data. Finally, the predicted results were integrated to generate a consensus gene set by EVidenceModeler (EVM) v. 1.1.1 [94] pipeline using the parameter of ‘Mode:STANDARD S-ratio: 1.13 score > 1000’, and PASA v. 2.0.2 [93] was run to modify and update gene untranslated region (UTR) and alternative splice variants to obtain more transcripts, with default parameters.

For non-coding RNAs prediction, Infenal 1.1 [95] was run to determine rRNAs and microRNAs according to the Rfam [96] and miRBase [97] databases using the parameter of 1e-5, while tRNAscan-SE v. 1.3.1 [98] was used to identify tRNAs employing default parameters. For pseudogene prediction, through GenBlastA v. 1.0.4 alignment [99], homologous gene sequences were searched on the genome after shielding the true gene loci, using the parameter of ‘-e 1e-5’. Then, GeneWise v. 2.4.1 [100] was run to search for immature stop codons and frameshift mutation, using the parameter of ‘-both -pseudo’.

The functions of predicted genes in the assembled genome were identified based on the best matches across five databases using the threshold of -evalue 1e-5: Gene Ontology (GO) [101], Kyoto Encyclopedia of Genes and Genomes (KEGG) [102], eukaryotic ortholog groups (KOG) [103], Translation of the EMBL database (TrEMBL) [104], and Non-Redundant Protein Sequence database (NR).

### Phylogenetic reconstruction

To reconstruct the phylogenetic history of the Temminck’s tragopan, 11 other closely related Galliformes species with relatively good quality assembled genomes were selected, including one guineafowl (Numididae) species, one New World quail (Odontophoridae) species, and nine Phasianidae species, with detailed information listed in Table S6. The Numididae species (*Numidea meleagris*) was used as the outgroup. Orthofinder v. 2.4 [105] was employed to classify protein sequences. In total, 4,907 one-to-one orthologous genes were used for phylogenetic tree construction in IQ-TREE v. 1.6.11 [106]. Genes were firstly aligned using MAFFT v. 7.205 [107]. Gblocks v. 0.91b [108] was employed to remove the regions with poorly aligned scores or large difference areas with default parameters, and then the alignments were concatenated to form one supermatrix. Subsequently, the best substitution model (JTT + F + I + G4) was selected using ModelFinder [109], and the maximum likelihood (ML) method was used to reconstruct the phylogenetic tree with the bootstrap set to 1000.

### Divergence time estimation

The divergence time estimation of Galliformes species was based on the ML phylogenetic tree above. Divergence times were inferred by MCMCTree in the PAML v. 4.9i [110], with three calibration points retrieved from the TimeTree database [111] used for calibrating, including 6.83–13.4 MYA between *L. nycthemera* and *Chrysolophus pictus*, 27.5–37.5 MYA between *L. nycthemera* and *M. gallopavo*, and 41–52 MYA between *N. meleagris* and *Perdix hodgsoniae*. The parameters of gradient and Hessian were used. Using the ML method, the correlated molecular clock and JC69 model were run to estimate divergence times. Two repeated calculations were performed to observe the consistency (value = 1). The parameters were set as burnin of 5 000 000, sampfreq of 30, and nsample of 10 000 000.

### Synteny analysis

The genome assembly of the Temminck’s tragopan was aligned against the chicken genome assemble result (GRCg6a) using diamond v. 0.9.29.130 [112] to determine similar gene pairs with e value less than 1e − 5 and C score greater than 0.5. MCScanX [113] was used to estimate neighboring links on chromosomes of similar gene pairs with default parameters. JCVI [114] was run to display large-scale synteny blocks.

### Gene family expansion and contraction

The expansion and contraction of gene families were performed among 12 sampled Galliformes species to understand the evolutionary dynamics of genes. These species were analyzed using CAFE v. 4.2 [115] based on the phylogenetic tree with divergence times and gene family cluster results. A random birth and death process model was used, and both the family-wide *P*-Values and viterbi *P*-Values less than 0.05 were used to detect significantly expanded or contracted gene families of each lineage on the phylogenetic tree. The expansion of gene families of the Temminck’s tragopan were annotated using PANTHER V. 15 [116] with default parameters. Functional enrichment analyses were further performed on the expanded and contracted gene families using clusterProfile v3.14.0 [117].

### Positive selection analysis

With the phylogenetic tree, one-to-one orthologous genes of five closely related Galliformes species including the Tibetan partridge, wild turkey, Reeves’s pheasant, Indian peafowl, and chicken were retrieved to identify potential positively selected genes (PSGs). MAFFT v. 7.205 [107] was used to align protein sequences of each gene family, and the corresponding coding sequence alignments were back-translated from protein alignments employing PAL2NAL [118]. The branch-site model of CodeML in PAML [110] was used to detect PSGs, with the Temminck’s tragopan set as the foreground branch and other species performed as the background branch. Two models, model A, and null model, were run using parameters of model = 2 and fix_kappa = 0, and the likelihood ratio tests (LRTs) were then employed to compare these two models using the chi2 program. Genes with *P* values less than 0.05 was considered to be significant differences. Furthermore, the posterior probability (PP) was obtained using a Bayes Empirical Bayes (BEB) method, and genes with a *PP* value greater than 0.95 was defined as significant PSGs. Finally, functional enrichment analysis of identified PSGs was performed via clusterProfile v3.14.0 [117].

### Supplementary Information

**Additional file 1: Fig. S1.** The gene predicting and contamination assessing result. a gene predicting result from three methods. b contamination assessment.

**Additional file 2: Fig. S2.** Phylogenetic evolution of 12 sampled Galliformes species. a gene family cluster. b phylogenetic tree. c divergence time.

**Additional file 3: Fig. S3.** Genome synteny between the Temminck’s tragopan and the chicken.

**Additional file 4: Fig. S4.** GO enrichment results in the Temminck's tragopan. a expanded gene families, b PSGs.

**Additional file 5: Fig. S5.** Top 10 significant GO and KEGG terms of PSGs in the Temminck's tragopan. a cellular component in the GO database. b molecular function in the GO database. c biological process of the GO database. d the KEGG database.

**Additional file 6: Table S1.** The sequencing data based on Illumina platform in the Temminck’s tragopan. **Table S2.** Repeat sequences in the Temminck’s tragopan. **Table S3.** The main terms in three GO categories corresponding to Fig. 2a and Fig. S4. **Table S4.** Identified pathways and genes related with plumage color and feather development, immune response, and energy metabolism in PSGs in the Temminck’s tragopan. **Table S5.** 39 genes enriched in the MAPK signaling pathway (ko04010) in PSGs in the Temminck’s tragopan. **Table S6.** 12 sampled Galliformes species used for comparative genomic analyses.

## Data Availability

The sequence reads of the Temminck’s tragopan genome were deposited to the National Center for Biotechnology Information (NCBI) database with accession number of BioProject (PRJNA1000723), BioSample (SAMN36776254), and Sequence Read Archive (SRA) (SRR25487939 and SRR25487940), respectively.

## Abbreviations

BP: Biological process
BUSCO: Benchmarking universal single-copy orthologs
CC: Cellular component
CEGMA: Core eukaryotic genes mapping approach
GO: Gene ontology
HPD: Highest posterior density
KEGG: Kyoto encyclopedia of genes and genomes
KOG: Eukaryotic ortholog groups
LINEs: Long interspersed nuclear elements
MF: Molecular function
MYA: Million years ago
NR: Non-redundant protein sequence database
PSGs: Positively selected genes
SINEs: Short interspersed nuclear elements
TEs: Transposable elements
TrEMBL: Translation of the EMBL database
UCEs: Ultraconserved elements
UTR: Untranslated region
