# Supplementary figures and images for: The draft genome of the Temminck’s tragopan (Tragopan temminckii) with evolutionary implications
Source: BMC Genomics. 2023 Dec 7;24:751. doi: 10.1186/s12864-023-09857-6 (PMC10702090; doi:10.1186/s12864-023-09857-6)

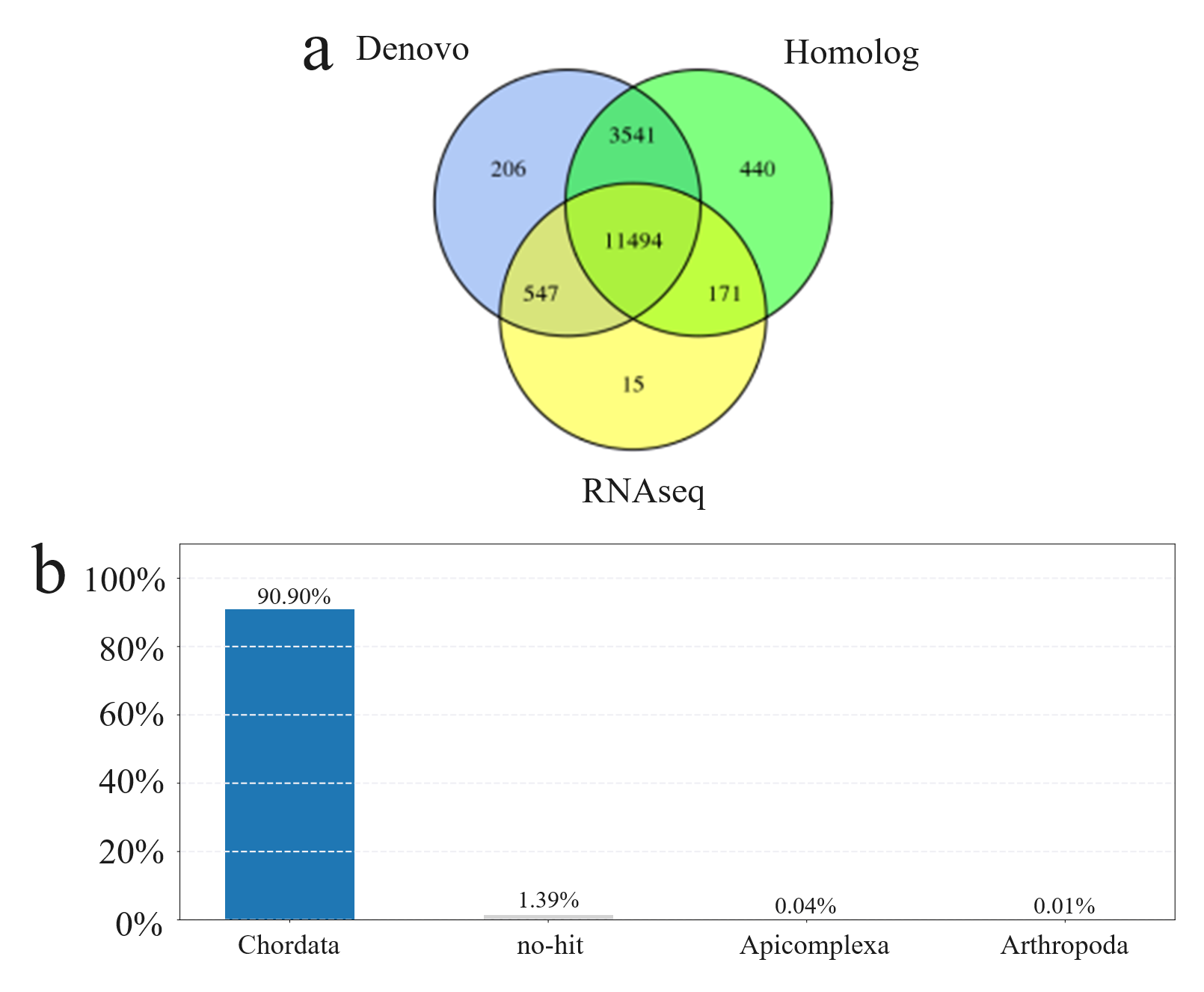

Supplement: Supplementary file 1 — Additional file 1: Fig. S1. The gene predicting and contamination assessing result. a gene predicting result from three methods. b contamination assessment. [file 12864_2023_9857_MOESM1_ESM.png]

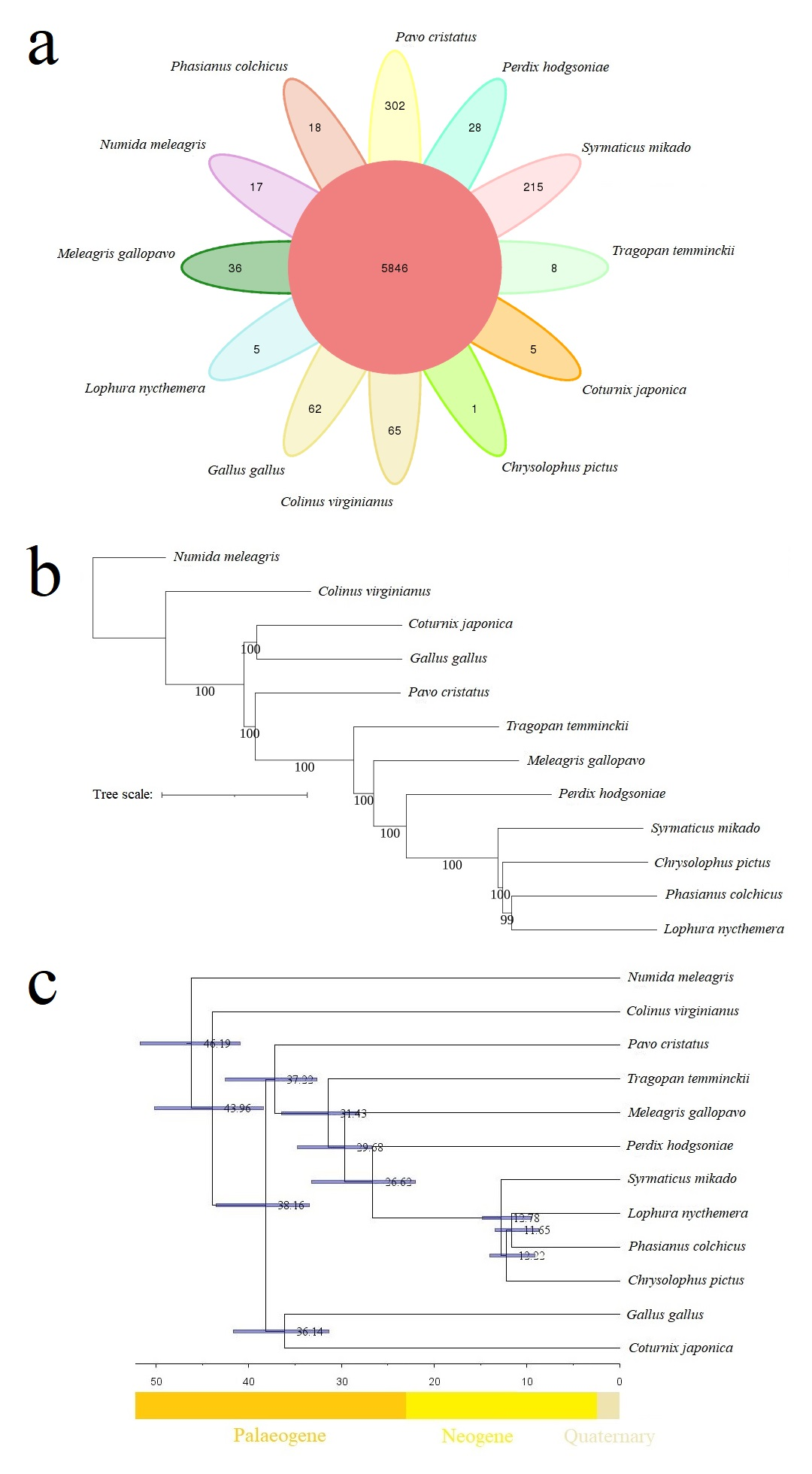

Supplement: Supplementary file 2 — Additional file 2: Fig. S2. Phylogenetic evolution of 12 sampled Galliformes species. a gene family cluster. b phylogenetic tree. c divergence time. [file 12864_2023_9857_MOESM2_ESM.png]

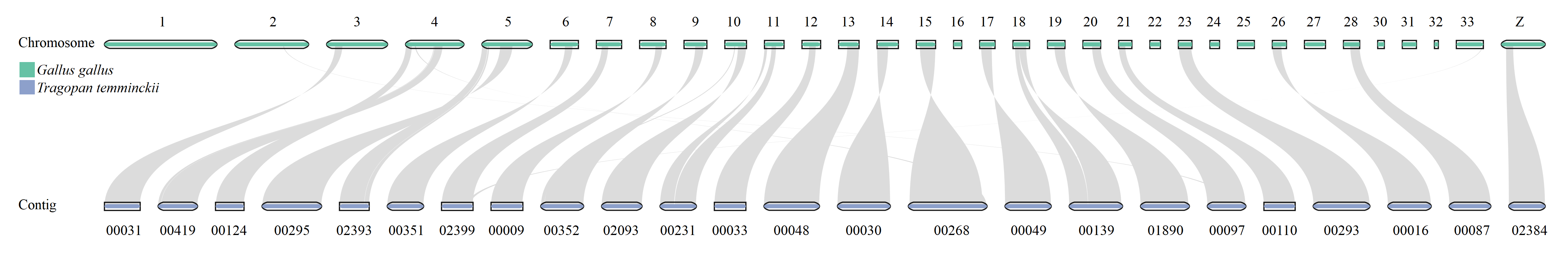

Supplement: Supplementary file 3 — Additional file 3: Fig. S3. Genome synteny between the Temminck’s tragopan and the chicken. [file 12864_2023_9857_MOESM3_ESM.png]

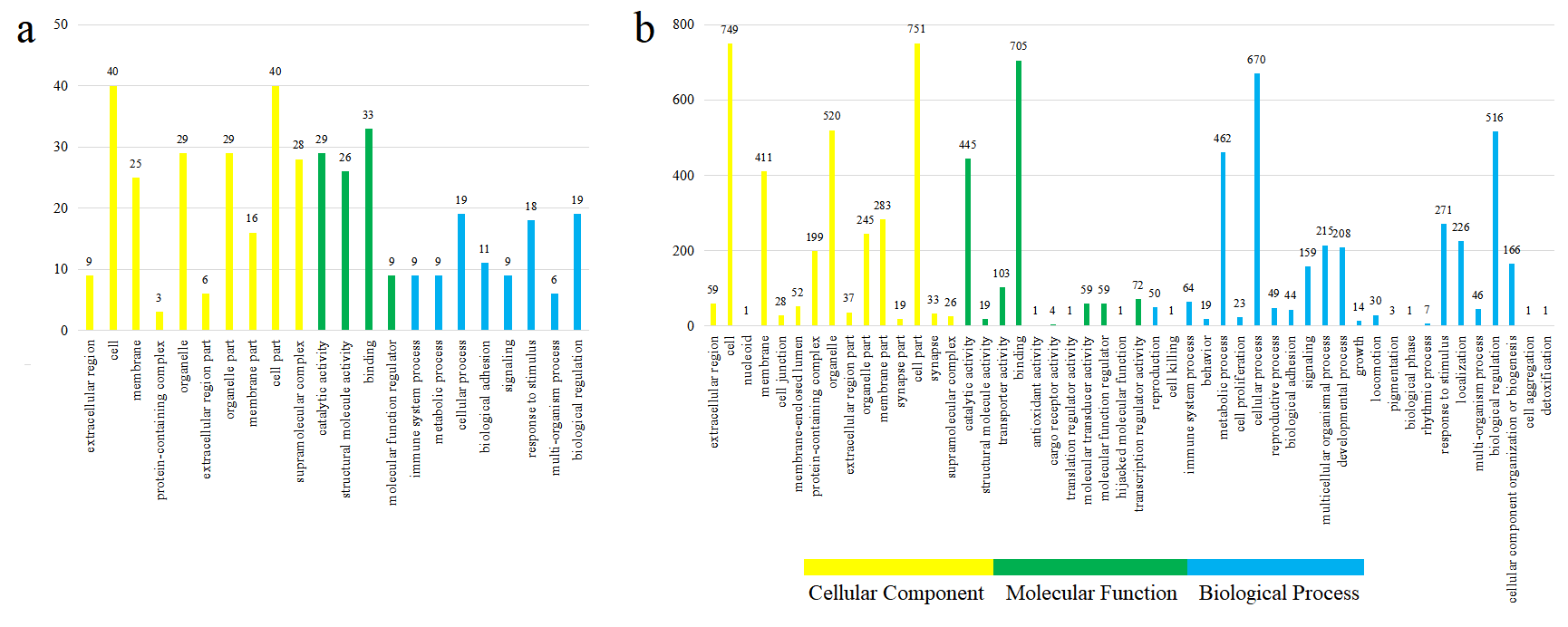

Supplement: Supplementary file 4 — Additional file 4: Fig. S4. GO enrichment results in the Temminck's tragopan. a expanded gene families, b PSGs. [file 12864_2023_9857_MOESM4_ESM.png]

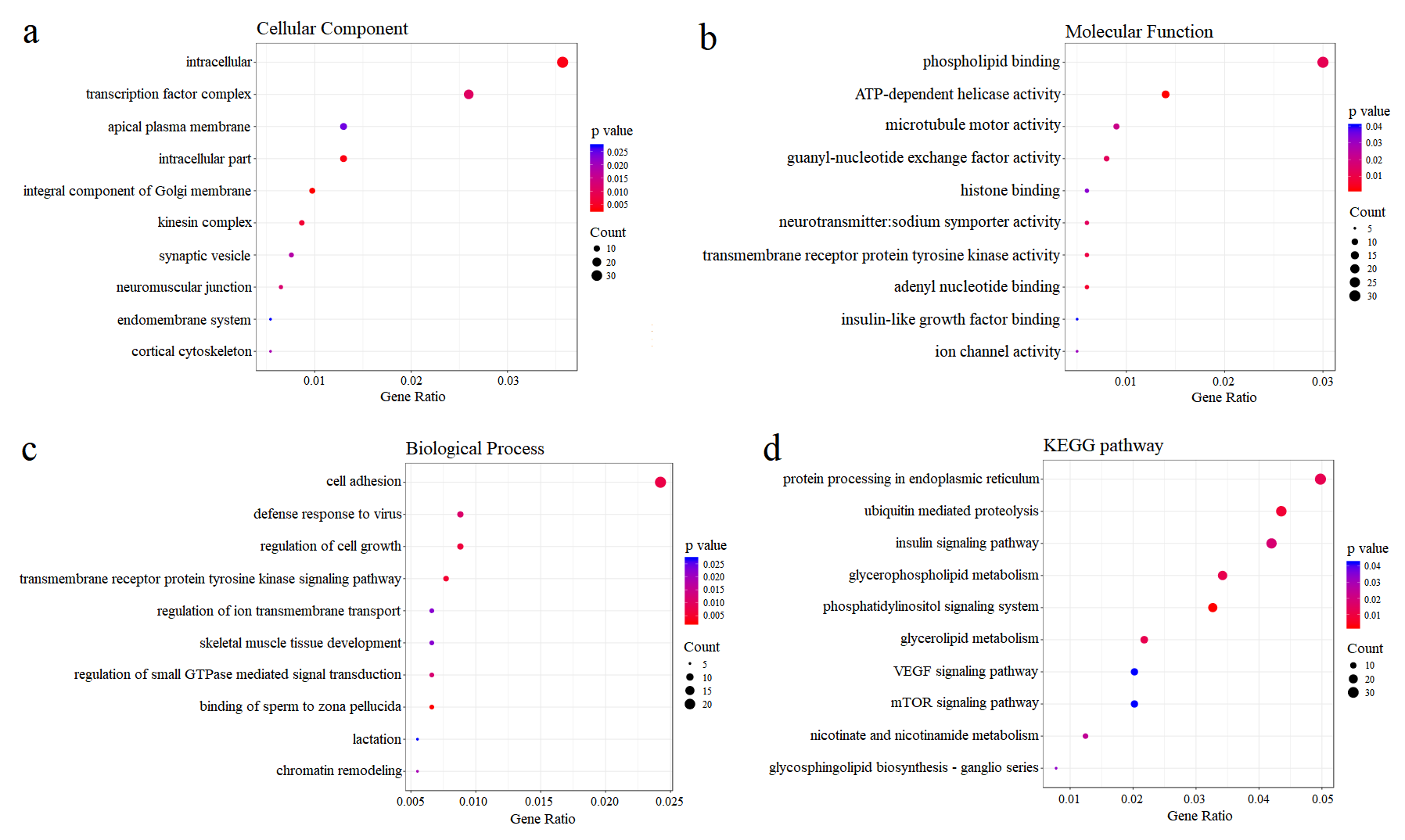

Supplement: Supplementary file 5 — Additional file 5: Fig. S5. Top 10 significant GO and KEGG terms of PSGs in the Temminck's tragopan. a cellular component in the GO database. b molecular function in the GO database. c biological process of the GO database. d the KEGG database. [file 12864_2023_9857_MOESM5_ESM.png]
