# Supplementary material for: The draft genome of the Temminck’s tragopan (Tragopan temminckii) with evolutionary implications
Source: BMC Genomics. 2023 Dec 7;24:751. doi: 10.1186/s12864-023-09857-6 (PMC10702090; doi:10.1186/s12864-023-09857-6)
Supplement: Supplementary file 6 — Additional file 6: Table S1. The sequencing data based on Illumina platform in the Temminck’s tragopan. Table S2. Repeat sequences in the Temminck’s tragopan. Table S3. The main terms in three GO categories corresponding to Fig. 2a and Fig. S4. Table S4. Identified pathways and genes related with plumage color and feather development, immune response, and energy metabolism in PSGs in the Temminck’s tragopan. Table S5. 39 genes enriched in the MAPK signaling pathway (ko04010) in PSGs in the Temminck’s tragopan. Table S6. 12 sampled Galliformes species used for comparative genomic analyses. [file 12864_2023_9857_MOESM6_ESM.docx]

**Supplementary Information**

**Fig. S1** The gene predicting and contamination assessing result. **a** gene predicting result from three methods. **b** contamination assessment.

**Fig. S2** Phylogenetic evolution of 12 sampled Galliformes species. **a** gene family cluster. **b** phylogenetic tree. **c** divergence time.

**Fig. S3** Genome synteny between the Temminck's tragopan and the chicken.

**Fig. S4** GO enrichment results in the Temminck's tragopan. **a** expanded gene families, **b** PSGs.

**Fig. S5** Top 10 significant GO and KEGG terms of PSGs in the Temminck's tragopan. **a** cellular component in the GO database. **b** molecular function in the GO database. **c** biological process of the GO database. **d** the KEGG database.

**Table S1** The sequencing data based on Illumina platform in the Temminck's tragopan.

| Library | Data (Gb) | Depth (×) | Q20 (%) | Q30 (%) |
| --- | --- | --- | --- | --- |
| 270 bp_1 | 14.85 | 14.01 | 96.64 | 92.34 |
| 270 bp_2 | 17.34 | 16.36 | 96.75 | 92.51 |
| 270 bp_3 | 17.54 | 16.55 | 97.19 | 93.12 |
| 350 bp_1 | 21.90 | 20.66 | 96.78 | 92.80 |
| 350 bp_2 | 22.08 | 20.83 | 96.87 | 93.00 |
| Total | 93.71 | 88.41 | --- | --- |

**Table S2** Repeat sequences in the Temminck's tragopan.

| Type | Number | Length | Rate (%) |
| --- | --- | --- | --- |
| Class I/DIRS | 612 | 41705 | 0 |
| Class I/LINE | 294632 | 86375153 | 8.11 |
| Class I/LTR | 53389 | 17768749 | 1.67 |
| Class I/LTR/Copia | 551 | 146056 | 0.01 |
| Class I/LTR/Gypsy | 6092 | 896143 | 0.08 |
| Class I/PLE\|LARD | 58285 | 11931558 | 1.12 |
| Class I/SINE | 1517 | 201780 | 0.02 |
| Class I/TRIM | 1452 | 766818 | 0.07 |
| Class I/Unknown | 350 | 31377 | 0 |
| Class II/Crypton | 154 | 9708 | 0 |
| Class II/Helitron | 752 | 62112 | 0.01 |
| Class II/MITE | 53 | 25189 | 0 |
| Class II/Maverick | 523 | 46623 | 0 |
| Class II/TIR | 40132 | 10305427 | 0.97 |
| Class II/Unknown | 4933 | 424227 | 0.04 |
| Potential Host Gene | 1247 | 551631 | 0.05 |
| SSR | 2313 | 1685298 | 0.16 |
| Unknown | 15405 | 4600565 | 0.43 |
| Total without overlap: | 482392 | 117223727 | 11 |

**Table S3** The main terms in three GO categories corresponding to Fig. 2a and Fig. S4.

| Term | Gene number | | |
| --- | --- | --- | --- |
|  | Genome | Expanded gene family | PSG |
| Cellular Component |  |  |  |
| cell part (GO:0005623) | 5813 | 40 | 751 |
| cell (GO:0044464) | 5804 | 40 | 749 |
| Molecular Function |  |  |  |
| binding (GO:0005488) | 5232 | 33 | 705 |
| catalytic activity (GO:0003824) | 3166 |  |  |
| Biological Process |  |  |  |
| cellular process (GO:0009987) | 5145 | 19 | 670 |
| single-organism process (GO:0044699) | 4477 |  |  |
| biological regulation (GO:0065007) |  | 19 |  |
| response to stimulus (GO:0050896) |  | 18 |  |

**Table S4** Identified pathways and genes related with plumage color and feather development, immune response, and energy metabolism in PSGs in the Temminck's tragopan.

| Item | Accession number | Term |
| --- | --- | --- |
| GO enrichment |  |  |
| plumage color and feather development | Biological Process |  |
|  | GO:0030318 | melanocyte differentiation |
|  | GO:0031069 | hair follicle morphogenesis |
|  | GO:0001942 | hair follicle development |
|  | GO:0030216 | keratinocyte differentiation |
| immune response | Biological Process |  |
|  | GO:0051607 | defense response to virus |
|  | GO:0046677 | response to antibiotic |
|  | GO:0050853 | B cell receptor signaling pathway |
|  | GO:0034644 | cellular response to UV |
|  | GO:0000077 | DNA damage checkpoint |
|  | GO:0031572 | G2 DNA damage checkpoint |
|  | GO:0048010 | vascular endothelial growth factor receptor signaling pathway |
|  | GO:0008543 | fibroblast growth factor receptor signaling pathway |
|  | GO:0019221 | cytokine-mediated signaling pathway |
|  | GO:0006281 | DNA repair |
|  | GO:0050727 | regulation of inflammatory response |
|  | GO:0034154 | toll-like receptor 7 signaling pathway |
|  | GO:0009636 | response to toxic substance |
|  | GO:0006897 | endocytosis |
|  | GO:0006974 | cellular response to DNA damage stimulus |
|  | GO:0016055 | Wnt signaling pathway |
|  | GO:0007219 | Notch signaling pathway |
|  | GO:0002755 | MyD88-dependent toll-like receptor signaling pathway |
|  | GO:0009408 | response to heat |
|  | GO:0010212 | response to ionizing radiation |
|  | GO:0035682 | toll-like receptor 21 signaling pathway |
|  | GO:0001666 | response to hypoxia |
|  | GO:0006950 | response to stress |
|  | GO:0007179 | transforming growth factor beta receptor signaling pathway |
|  | GO:0042493 | response to drug |
|  | GO:0035681 | toll-like receptor 15 signaling pathway |
|  | GO:0050830 | defense response to Gram-positive bacterium |
|  | GO:0050896 | response to stimulus |
|  | GO:0043401 | steroid hormone mediated signaling pathway |
|  | GO:0034134 | toll-like receptor 2 signaling pathway |
|  | GO:0034142 | toll-like receptor 4 signaling pathway |
|  | GO:0034146 | toll-like receptor 5 signaling pathway |
|  | GO:0030509 | BMP signaling pathway |
|  | GO:0006289 | nucleotide-excision repair |
|  | GO:0042832 | defense response to protozoan |
|  | GO:0009615 | response to virus |
|  | GO:0006302 | double-strand break repair |
|  | GO:0034138 | toll-like receptor 3 signaling pathway |
|  | GO:0071456 | cellular response to hypoxia |
|  | GO:0042060 | wound healing |
|  | GO:0006954 | inflammatory response |
|  | GO:0006955 | immune response |
|  | GO:0042742 | defense response to bacterium |
|  | GO:0006816 | calcium ion transport |
|  | GO:0050852 | T cell receptor signaling pathway |
|  | GO:0006979 | response to oxidative stress |
|  | Molecular Function |  |
|  | GO:0042393 | histone binding |
|  | GO:0004707 | MAP kinase activity |
|  | GO:0005154 | epidermal growth factor receptor binding |
|  | GO:0003707 | steroid hormone receptor activity |
|  | GO:0017017 | MAP kinase tyrosine/serine/threonine phosphatase activity |
|  | GO:0017134 | fibroblast growth factor binding |
|  | GO:0005509 | calcium ion binding |
| energy metabolism | Biological Process |  |
|  | GO:0006869 | lipid transport |
|  | GO:0006629 | lipid metabolic process |
|  | GO:0055114 | oxidation-reduction process |
|  | GO:0006096 | glycolytic process |
|  | GO:0007005 | mitochondrion organization |
|  | GO:0045454 | cell redox homeostasis |
|  | GO:0008152 | metabolic process |
|  | GO:0016042 | lipid catabolic process |
|  | GO:0006635 | fatty acid beta-oxidation |
|  | GO:0015986 | ATP synthesis coupled proton transport |
|  | GO:0006633 | fatty acid biosynthetic process |
|  | Cellular Component |  |
|  | GO:0005777 | peroxisome |
|  | GO:0005739 | mitochondrion |
|  | Molecular Function |  |
|  | GO:0050661 | NADP binding |
|  | GO:0016887 | ATPase activity |
|  | GO:0008060 | GTPase activator activity |
|  | GO:0070403 | NAD+ binding |
|  | GO:0016702 | oxidoreductase activity, acting on single donors with incorporation of molecular oxygen, incorporation of two atoms of oxygen |
|  | GO:0042626 | ATPase activity, coupled to transmembrane movement of substances |
|  | GO:0019003 | GDP binding |
|  | GO:0008137 | NADH dehydrogenase (ubiquinone) activity |
|  | GO:0016773 | phosphotransferase activity, alcohol group as acceptor |
|  | GO:0051287 | NAD binding |
|  | GO:0016491 | oxidoreductase activity |
|  | GO:0003924 | GTPase activity |
|  | GO:0005525 | GTP binding |
| KEGG enrichment |  |  |
| plumage color | ko04916 | melanogenesis |
| immune response | ko04120 | Ubiquitin mediated proteolysis |
|  | ko04150 | mTOR signaling pathway |
|  | ko04370 | VEGF signaling pathway |
|  | ko04012 | ErbB signaling pathway |
|  | ko04144 | Endocytosis |
|  | ko04350 | TGF-beta signaling pathway |
|  | ko03430 | mismatch repair |
|  | ko04145 | Phagosome |
|  | ko04010 | MAPK signaling pathway |
|  | ko00100 | Steroid biosynthesis |
|  | ko03420 | Nucleotide excision repair |
|  | ko04310 | Wnt signaling pathway |
|  | ko04330 | Notch signaling pathway |
|  | ko04068 | FoxO signaling pathway |
|  | ko04142 | Lysosome |
|  | ko04630 | Jak-STAT signaling pathway |
|  | ko00983 | Drug metabolism - other enzymes |
|  | ko04620 | Toll-like receptor signaling pathway |
|  | ko03320 | PPAR signaling pathway |
|  | ko04622 | RIG-I-like receptor signaling pathway |
|  | ko04621 | NOD-like receptor signaling pathway |
|  | ko00140 | steroid hormone biosynthesis |
|  | ko04115 | p53 signaling pathway |
|  | ko00982 | Drug metabolism - cytochrome P450 |
|  | ko04060 | cytokine-cytokine receptor interaction |
|  | ko03410 | base excision repair |
|  | ko04140 | regulation of autophagy |
|  | ko04672 | Intestinal immune network for IgA production |
|  | ko04020 | Calcium signaling pathway |
|  | ko04514 | Cell adhesion molecules (CAMs) |
| energy metabolism | ko00071 | Fatty acid degradation |
|  | ko00061 | Fatty acid biosynthesis |
|  | ko00190 | Oxidative phosphorylation |
|  | ko00062 | Fatty acid elongation |
|  | ko01040 | Biosynthesis of unsaturated fatty acids |
|  | ko01212 | Fatty acid metabolism |

**Table S5** 39 genes enriched in the MAPK signaling pathway (ko04010) in PSGs in the Temminck's tragopan.

| Gene ID | Gene |
| --- | --- |
| EVMG000254.1 | *grb2* |
| EVMG000469.1 | *EGFR* |
| EVMG000561.1 | *MAPKAPK2* |
| EVMG000579.1 | *CACNA1S* |
| EVMG001918.1 | *FGF1* |
| EVMG002571.1 | *RRAS2* |
| EVMG002789.1 | *NFKB2* |
| EVMG005417.1 | *MAPK1* |
| EVMG005649.1 | *PPP3CA* |
| EVMG005689.1 | *MKNK2* |
| EVMG006769.1 | *MAPT* |
| EVMG007338.1 | *DUSP3* |
| EVMG007352.1 | *MAP4K4* |
| EVMG007938.1 | *TGFBR1* |
| EVMG008095.1 | *BRAF* |
| EVMG008323.1 | *RASGRP1* |
| EVMG008499.1 | *PLA2G4F* |
| EVMG008592.1 | *RAPGEF2* |
| EVMG008801.1 | *DUSP5* |
| EVMG009257.1 | *FGF20* |
| EVMG009349.1 | *MAPK8IP3* |
| EVMG009413.1 | *IL1RN* |
| EVMG010017.1 | *PPM1A* |
| EVMG010157.1 | *CACNA1I* |
| EVMG010257.1 | *CACNA1E* |
| EVMG010401.1 | *CACNA1C* |
| EVMG010408.1 | *PLA2G4A* |
| EVMG010550.1 | *CACNA1G* |
| EVMG010566.1 | *CHUK* |
| EVMG010602.1 | *TRAF2* |
| EVMG011248.1 | *CRK* |
| EVMG011269.1 | *Nlk* |
| EVMG011321.1 | *PDGFRA* |
| EVMG011801.1 | *MAP3K1* |
| EVMG012207.1 | *ATF2* |
| EVMG012742.1 | *MAPK14* |
| EVMG014037.1 | *MAP3K6* |
| EVMG014089.1 | *RASGRF2* |
| EVMG015093.1 | *PTPRR* |

**Table S6** 12 sampled Galliformes species used for comparative genomic analyses.

| Family | Species | Accession no. |
| --- | --- | --- |
| Numididae | *Numida meleagris* | GenBank (GCA_002078875.2) |
| Odontophoridae | *Colinus virginianus* | GenBank (GCA_000599465.2) |
| Phasianidae | *Chrysolophus pictus* | Ensembl (release-99) |
|  | *Coturnix japonica* | GenBank (GCA_001577835.1) |
|  | *Gallus gallus* | GenBank (GCA_000002315.5) |
|  | *Lophura nycthemera* | GenBank (GCA_021292215.1) |
|  | *Meleagris gallopavo* | GenBank (GCF_000146605.3) |
|  | *Pavo cristatus* | Ensembl (release-99) |
|  | *Perdix hodgsoniae* | Li et al. [37] |
|  | *Phasianus colchicus* | Ensembl (release-99) |
|  | *Syrmaticus mikado* | GenBank (GCA_003435085.1) |
|  | *Tragopan temminckii* | this study |
